# Coach leadership behavior and achievement goal orientation in relation to athlete engagement: the dual mediating role of basic psychological needs

**DOI:** 10.3389/fpsyg.2025.1527409

**Published:** 2025-03-31

**Authors:** Kaixin Li, Zhibu Cui, Tong Wang, Zhihua Li, Chengbo Yang

**Affiliations:** School of Sport and Training, Chengdu Sport University, Chengdu, China

**Keywords:** coach leadership behavior, achievement goal orientation, basic psychological needs, athlete engagement objective, athlete

## Abstract

**Objective:**

This study explored how coach leadership behavior, achievement goal orientation, and basic psychological needs affect athlete engagement.

**Methods:**

Based on self-determination theory, this study examined the influence of coach leadership behavior and achievement goal orientation on athlete engagement, as well as the mediating effect of basic psychological needs. The study utilized established scales, including the Leadership Behavior Scale for Coaches-15 (LSS-15), the Achievement Goal Questionnaire for Sport (AGQ-S), the Athlete Engagement Questionnaire (AEQ) and the Basic Psychological Needs Questionnaire (BPNQ).

**Results:**

A total of 351 valid questionnaires were analyzed. The results found that (1) basic psychological needs, coach leadership behavior, and achievement goal orientation can predict athlete engagement, and a correlation exists between them; (2) basic psychological needs play a double mediating role in coach leadership behavior and athlete engagement, achievement goal orientation, and athlete engagement. Coach leadership behavior and basic psychological needs have a chain mediation mechanism between achievement goal orientation and athlete engagement. Achievement goal orientation and basic psychological needs have a chain mediation mechanism between coach leadership behavior and athlete engagement; (3) after model testing and model difference comparison. The effect of the chain model based on the coach leadership behavior is more significant, indicating that the plasticity of the coach leadership behavior is the strongest.

**Conclusion:**

These findings analyze the relationship between coach leadership behavior, achievement goal orientation, basic psychological needs, and athlete engagement to optimize coach leadership behavior, guide the setting of reasonable achievement goal orientation, meet basic psychological needs, improve athlete engagement, and provide practical guidance for targeted intervention measures.

## 1 Introduction

Competitive sports, characterized by inherent competitiveness, challenges, and confrontations, inevitably expose athletes to sustained high-pressure conditions, predisposing them to burnout, mental fatigue, and related phenomena. Athlete burnout reflects an imbalance between personal resources and environmental demands, manifesting as immediate, transient physical and psychological stress reactions. Over time, these reactions accumulate due to ineffective recovery, evolving into a state of physical and psychological symptomatology and dysfunction, characterized by emotional/physical depletion, reduced sense of achievement, and negative sport evaluation. The prevention of athlete burnout represents a critical concern for both coaches and athletes (Ye et al., 2016b; Hodge et al., 2009). Athlete engagement, serving as an indicator of positive psychology, is advantageous in fostering positive attributes and alleviating negative psychological states in athletes (Ye et al., 2016a). The Sport Commitment Model, which serves as an early explanatory framework for athlete burnout, further proposes that the degree of personal dedication to physical activity can serve as an effective means of alleviating burnout among athletes (Scanlan et al., 1993; Schmidt and Stein, 1991). Athlete engagement can serve as a pivotal starting point for addressing athlete burnout, fostering athlete development and maturity, and establishing a solid foundation for enhanced athletic performance. Social cognitive theory points out that athlete engagement is driven by internal individual factors and external environmental factors (Young et al., 2014). In this context, the coach’s behavior, as the immediate leader of the sports team, serves as the primary external factor motivating athletes to engage actively in training and competition, thereby enhancing their athletic abilities. Individual factors can be divided into intelligence and non-intelligence factors, in all the non-intelligence factors, motivation is the most core element. Achievement goal orientation is a widely concerned motivational variable, mainly through the dynamic psychological process to fully express the impact of goals on athlete engagement, affecting the change of athletes’ sports performance and the achievement of competition goals (Ingrell et al., 2019). At present, most of the existing studies in the academic field only discuss the influence of coach leadership behavior, and achievement goal orientation on athlete engagement. Basic psychological needs are closely related to coaches’ leadership behavior, achievement goal orientation and athlete engagement, but the mediating mechanism of basic psychological needs is ignored. A single research perspective is always easy to fall into the binary isolation theory of either/or. In view of this, the research is based on the analysis of the relationship between the coach’s leadership behavior, achievement goal orientation, basic psychological needs, and athlete engagement perceived by high-level athletes, and the construction of an intermediary model for testing to explore the potential mechanism affecting athlete engagement and the influence difference from the internal and external support of individuals. The aim is to provide a theoretical basis for coaches and sports managers to promote athlete engagement level and to point out the direction for sports psychologists to effectively intervene in athlete engagement.

## 2 Literature review

### 2.1 Coach leadership behavior and athlete engagement

Coach leadership behaviors constitute a specific indicator of coaching quality, serving as a pivotal factor in enhancing sports performance and representing a significant research area within the field of sport science (Li et al., 2017). Its theoretical basis can be traced back to the multidimensional leadership model (Chelladurai, 1990), specifically, the five leadership behaviors—training and coaching, democracy, authoritarianism, social support, and positive feedback—manifest during the process of influencing athletes’ performance and behaviors (Chelladurai and Carron, 1983). Coach leadership behavior can exert a direct influence on athlete engagement. Coaches who exhibit greater social supportive behaviors towards their athletes can enhance athletes’ identification with training and competition, thereby fostering closer relationships between athletes and coaches (Wang, 2023). Research has also confirmed that there is a correlation between coaches paternalistic leadership behavior and athletes engagement, and that coaches’ benevolent leadership behavior and virtuous leadership behaviors have a positive impact on the relationship between athletes and coaches (Ma and Wang, 2006). On the other hand, coach leadership behavior can also have an indirect effect on athlete engagement by influencing other factors. Research shows that coaches’ paternalistic leadership correlates with athlete engagement, while benevolent and virtuous leadership behavior positively affects coach-athlete relationships. Additionally, coach leadership behavior can indirectly influence athlete engagement by affecting psychological resilience (Xu, 2022). Coach autonomy support can positively influence athlete engagement by influencing basic psychological needs and thus (De Francisco et al., 2018). The coach-athlete relationship partially mediates the effect of coach leadership behavior on athlete engagement (Gao et al., 2021). However, current research has pointed out that some coaches do not have a significant impact on athlete productivity, so the recruitment of excellent coaches is crucial to the development of athletes, coaches should consider how to develop their athletes during their tenure (Chris et al., 2023).

### 2.2 Achievement goal orientation and athlete engagement

Achievement goal orientation is rooted in achievement goal theory (Nicholls, 1984), which is an individual’s cognition of the purpose or reason for undertaking a task and his belief in completing the task. This construct possesses motivational, cognitive, affective, and behavioral characteristics (Es and Cs, 1988) and emphasizes the differentiation of motivation between mastery and performance goals. In the field of education, the relationship between achievement goal orientation, academic engagement (Shida, 2024), and academic performance (Xu et al., 2024) has been widely discussed; in the field of sports, self-determination theory states that a hierarchical model of motivation distinguishes between intrinsic motivation (interest-driven) and extrinsic motivation (reward-driven) (Vallerand, 2000). Therefore, the achievement goal orientation can be regarded as the internal factor that affects athlete engagement. Martin’s research highlights athlete engagement as crucial for understanding athletes’ internal drive and motivation. It suggests motivation increases when athletes aim to master their sport (Amechi and Estera, 2017). Jung et al. (2021) found a significant positive correlation between sports participation motivation and achievement goal orientation among high school basketball players, with the latter notably influencing achievement behavior. Specifically, motivation related to technical skill development and achieving a sense of accomplishment, recreation, and health positively influenced task goal orientation (Jung et al., 2021). When Martins et al. (2017) assessed the relationship between athletes’ motivation, engagement, and personal and social responsibility, the validation yielded that task orientation in achievement goal orientation was positively correlated with athlete engagement.

### 2.3 The mediating role of basic psychological needs

Self-Determination Theory (SDT) (Ryan and Deci, 2000) is a central framework for understanding athlete engagement. It emphasizes the satisfaction of basic psychological needs as a key prerequisite for optimal athlete functioning. Basic needs theory, a subset of self-determination theory, encompasses the concepts of autonomy, competence, and relational needs. Autonomy needs are an individual’s perceived power to make choices and decisions; competence needs are an individual’s perceived sense of competence when interacting with the environment; and relational needs are an individual’s perceived sense of belonging in the surrounding environment (Huo et al., 2011). The study of basic psychological needs has received extensive attention in the field of sport, and a large number of studies have confirmed the influence of basic psychological needs on the factors involved. Quested and Duda’s findings suggest that psychological needs are mediators of motivational climate with positive and negative affect (Quested and Duda, 2009). Chinese scholars, using domestic athletes as research subjects, concluded that basic psychological needs and autonomy motivation play a partially mediating role between coaches’ autonomy support and athletes’ psychological fatigue, and the chain mediation of basic psychological need → autonomy motivation plays a fully mediating role (Liu et al., 2023). Cai et al. (2020) demonstrated that basic psychological need satisfaction partially mediated between virtuous leadership behaviors and commitment while playing a fully mediating role between benevolent leadership behaviors and athlete commitment. Cheng and Cheng (2015) explored the moderating role of the learning environment’s support for college students’ sense of connectedness, autonomy, and competence in the relationship between achievement goals and subjective well-being. It was pointed out that under high levels of support for relatedness and autonomy, college students who adopted mastery-trend goals and performance-trend goals experienced strong positive emotions; low levels of support for basic psychological needs favored the adoption of performance-avoidance goals for students to experience positive emotions (Cheng and Cheng, 2015). Zhu et al. (2011) suggested that basic needs theory is applicable among Chinese athletes and that competence needs and relational needs are mediators of coach autonomy auspices and fatigue and training satisfaction, but the manifestations are not quite consistent with Western findings.

### 2.4 A study of the relationship between coach leadership behavior, achievement goal orientation, basic psychological needs, and athlete engagement

Coach leadership behavior and achievement goal orientation as extrinsic and intrinsic factors affecting athlete engagement. Firstly, extrinsic factors affecting athlete engagement, Ratelle CF, Larose S, and Guay F’s study showed that autonomy support from coaches can satisfy two basic psychological needs of athletes, autonomy and interpersonal relationships, which can increase the level of athlete engagement. A social environment that supports autonomy and emphasizes progress and endeavor may help to maximize the fulfillment of athletes’ basic needs, which in turn may contribute to the well-being of youth sport participants (Reinboth et al., 2004). In addition, studies have pointed out that currently more athletes have the identity of student roles, and if coaches as influencers further complicate the conflict between their athlete and student roles, athletes may have greater psychological and sociological consequences (Fridley et al., 2024). Conversely, there are intrinsic factors that influence athlete engagement. The combination of achievement goal theory and self-determination theory was employed to identify the specific factors that can optimize an individual’s affective, cognitive, and behavioral engagement in achievement situations (Diseth et al., 2012; Benita et al., 2014). In the exploration of the antecedents of athlete engagement, scholars have suggested that self-determination theory is considered a potential basis for studying the antecedents of athlete engagement. It has been hypothesized that the ‘fulfillment’ of basic psychological needs may be a motivational precursor to athlete engagement (Lonsdale et al., 2007). Using a sample of outstanding athletes, Hodge et al. (2009) tested the partial mediating relationship and the extent of the effect of athlete engagement on the relationship between basic psychological needs and motivational tendencies, i.e., need satisfaction positively predicted athlete engagement, and need satisfaction and athlete engagement jointly predicted motivational tendencies. Burnout as the antithesis of commitment, many foreign scholars have studied the relationship between the two. Alvarado et al. (2016) analyzed the relationship between basic motivational needs, burnout, and career commitment of football players, and its results showed that the three basic psychological needs had a significant positive predictive effect on the commitment factors of the athletes, and a significant negative predictive effect on the burnout symptoms of the athletes. Where basic psychological needs are perceived to be satisfied, subjective happiness and well-being increase, and autonomy perception is a significant predictor of burnout and engagement factors (Alvarado et al., 2016). Xu Jinpeng et al. artificially explored the relationship between adolescents’ basic psychological needs and sports motivation on sports enthusiasm. Sports motivation can largely affect the basic psychological needs of adolescents, and sports motivation through the degree of satisfaction of the basic psychological needs will produce different sports enthusiasm, which in turn promotes adolescents’ participation in sports (Xu et al., 2022).

Based on the above theoretical analysis and empirical research, coaches’ leadership behavior, achievement goal orientation, athletes basic psychological needs, and athlete engagement are closely related, and coach behavior and achievement goal orientation may contribute to the level of athlete engagement by achieving the satisfaction of the basic psychological needs of the perpetrators of the behaviors. Based on this, this study proposes the following model hypotheses: H1 Basic psychological needs may have a dual mediating role in coach leadership behavior and athlete engagement and achievement goal orientation and athlete engagement; H2 There may be a chain mediating mechanism between achievement goal orientation and athlete engagement for coach leadership behavior and basic psychological needs; H3 Achievement goal orientation and basic psychological needs may have a chain mediating mechanism between coach leadership behavior and athlete engagement.

## 3 Research objects and methods

### 3.1 Research subjects

Adopting the whole group sampling method from Sichuan Province, Guangdong Province, Jiangsu Province, Liaoning Province, Shandong Province, and other provinces and municipalities of local training teams, a number of professional sports teams of high-level athletes (all level 1 and above athletes) were subjects. The questionnaire used a paper version of the questionnaire and the network survey of the two distributions. A total of 376 questionnaires were issued, and 351 valid questionnaires were recovered, with an effective recovery rate of 93.35%.

Ethics committee approval was obtained from the college, in line with the Declaration of Helsinki (Emanuel et al., 2008), and all vocational college students were informed about the study’s purpose prior to the survey. Participants were assured that their personal information and responses would be kept confidential and used exclusively for research purposes. Among them, there were 218 males and 133 females; 58 fitness generals and 293 Grade 1 athletes; 184 in collective events and 167 in individual events.

The R language program developed by Schoemann et al. (2017) based on Monte Carlo simulation is used in this study. With power = 0.8, a Monte Carlo sample size of 20,00 per replicate, and a confidence level of 95%, the analysis showed a lower limit of 139 participants. To ensure potential data omissions or deficiencies, 351 athletes were included in the study.

### 3.2 Research tools

#### 3.2.1 Leadership behavior scale

The Leadership Behavior Scale for Coaches was adapted from the Leadership Scale for Sports (LSS) developed by Chelladurai and Saleh (1980) and further refined based on the Leadership Behavior Scale for Coaches-15 by Teques et al. (2021). Due to cultural and linguistic differences, the transplantation and adaptation of this scale pose significant validity concerns and necessitate ensuring and enhancing the equivalence of measurement instruments (Epstein et al., 2015). Consequently, the Coaches’ Leadership Behavior Scale-15 underwent local revision. This scale encompassed 15 items across five dimensions: training and coaching behavior, democratic behavior, authoritarian behavior, social support behavior, and positive feedback behavior. It employed a 5-point Likert scale, ranging from 1 (‘never’) to 5 (‘always’). The Cronbach’ s alpha coefficient for the overall questionnaire was 0.906, with subscale Cronbach’ s alpha coefficients ranging from 0.790 to 0.855 across the five dimensions. The results of the confirmatory factor analysis indicated good construct validity of the questionnaire, with fit indices including CMIN/DF = 1.774, RMSEA = 0.047, NFI = 0.949, RFI = 0.949, CFI = 0.977, IFI = 0.977, and TLI = 0.970.The AVE value is equal to 0.63, the CR value is equal to 0.96, and the convergence validity is achieved.

#### 3.2.2 Achievement goal questionnaire

The Achievement Goal Questionnaire for Sport was developed by Conroy et al. (2010) and revised by Zhu (2010). The questionnaire consists of four dimensions: mastery-approach, mastery-avoidance, performance-approach, and performance-avoidance, with a total of 12 items. It uses a 5-point rating scale, ranging from 1 (‘never’) to 5 (‘always’). The Cronbach’s *α* coefficient of the total questionnaire is 0.890, and the Cronbach’s α coefficients of the four dimensions are 0.845, 0.816, 0.842, and 0.823. The results of the confirmatory factor analysis are CMIN/DF = 1.313, RMSEA = 0.030, GFI = 0.972, RFI = 0.958, CFI = 0.992, IFI = 0.993, and TLI = 0.990, indicating that the questionnaire has good structural validity. The AVE value is equal to 0.62, the CR value is equal to 0.95, and the convergence validity is good.

#### 3.2.3 Athlete engagement questionnaire

The Athlete Engagement Questionnaire (AEQ) was developed by Lonsdale et al. (Zhu, 2010). The AEQ has 16 entries comprising four dimensions, namely, confidence, dedication, energy and enthusiasm. A 5-point Likert-type scale was used, ranging from 1 (‘never’) to 5 (‘always’). The Cronbach’s alpha coefficient for the total questionnaire was 0.921, 0.870 for the self-confidence dimension, 0.867 for the vitality dimension, 0.851 for the dedication dimension, and 0.838 for the enthusiasm dimension. The results of the confirmatory factor analysis are CMIN/DF = 1.504, RMSEA = 0.038, GFI = 0.951, RFI = 0.941, CFI = 0.983, IFI = 0.983, and TLI = 0.980, indicating that the questionnaire had good structural validity. The AVE value is equal to 0.60, the CR value is equal to 0.96, and the convergence validity is good.

#### 3.2.4 Basic psychological needs questionnaire

The Basic Psychological Needs Questionnaire was developed by Vlachopoulos and Michailidou (2006). The questionnaire consists of 12 items divided into three dimensions: autonomy needs, competence needs, and relationship needs, and is rated on a 5-point scale ranging from 1 (‘never’) to 5 (‘always’). The Cronbach’ s alpha coefficient for the total questionnaire was 0.906, and for the three dimensions, it was 0.875, 0.838, and 0.849, respectively. The results of the validated factor analyses were CMIN/DF = 1.252, RMSEA = 0.027, GFI = 0.963, RFI = 0.952, CFI = 0.992, IFI = 0.992, and TLI = 0.990, indicating that the structural validity of the questionnaire was good. The AVE value is equal to 0.56, and the CR value is equal to 0.94, reaching the convergence level.

### 3.3 Statistical analysis

Utilizing SPSS 27.0 and AMOS 27.0 software, the study initially conducted exploratory and confirmatory factor analyses on the Coaches’ Leadership Behavior Scale, Achievement Goal Orientation Scale, Basic Psychological Needs Scale, and Athlete Engagement Scale. These analyses aimed to extract the principal components of the revised scales and to assess the questionnaires’ reliability and validity. Secondly, descriptive statistical analysis and demographic difference analysis were conducted for the four variables, as shown in Table 1, and univariate and multivariate normality hypotheses were tested for all observed variables. Univariate normality was evaluated by calculating the skewness and kurtosis of each observed variable: the absolute skewness values of all items were <2 (range: −0.830 to 0.804), and the absolute kurtosis values were <7 (range: −1.026 to 0.096), meeting the standard of univariate normality. Multivariate normality was evaluated by the Mardia test implemented in the R-package MVN, which showed a normalized kurtosis value of −0.82, indicating that the data conformed to a multivariate normal distribution (Yuan et al., 2005). The multicollinearity problem in the modeling process was evaluated by variance inflation factor. Thirdly, Pearson correlation analysis was used to explore the relationship between coaches’ leadership behavior, achievement goal orientation, basic psychological needs, and athlete engagement, and the Harman single factor test was used to clarify the common method bias among variables. Finally, the maximum likelihood method was used to fit the path coefficients of the model, and the bias-corrected bootstrap2000 confidence interval estimation method was used to test the mediation effect, and the confidence level was set to 95%.

**Table 1:** Mean, standard deviation, and Pearson’s correlation coefficient between variables (*n* = 351).

Variant	*M*	SD	1	2	3	4	5	6	7	8	9	10	11	12	13	14	15	16
1. Training and coaching behavior	3.71	0.91	**0.56**															
2. Democratic behavior	3.71	1.05	0.54**	**0.67**														
3. Authoritarian behavior	3.23	1.08	−0.45**	−0.46**	**0.67**													
4. Social support behavior	3.58	1.03	0.50**	0.39**	−0.53**	**0.66**												
5. Positive feedback behavior	3.90	0.99	0.45**	0.38**	−0.49**	0.48**	**0.61**											
6. Mastery-convergent goal orientation	3.71	1.03	0.21**	0.15**	−0.13**	0.28**	0.20**	**0.65**										
7. Mastery-avoidance targeting	2.40	0.87	−0.26**	−0.19**	0.23**	−0.38**	−0.21**	−0.51**	**0.82**									
8. Achievement-convergence goal orientation	3.76	0.91	0.20**	0.18**	−0.22**	0.25**	0.27**	0.46**	0.49**	**0.84**								
9. Achievement-avoidance targeting	2.30	0.98	−0.17**	−0.18	0.19**	−0.34**	−0.26**	−0.49**	0.50**	−0.40**	**0.82**							
10. Need for autonomy	3.59	0.76	0.33**	0.17**	−0.21**	0.35**	0.17**	0.35**	−0.32**	0.29**	−0.28**	**0.58**						
11. Competency needs	3.79	0.75	0.23**	0.18**	−0.34**	0.29**	0.21**	0.31**	−0.27**	0.31**	−0.29**	0.45**	**0.57**					
12. Need for belonging	3.72	0.74	0.31**	0.19**	−0.36**	0.42**	0.25**	0.31**	−0.19**	0.36**	−0.42**	0.57**	0.56**	**0.53**				
13. Confidence	3.79	0.94	0.35**	0.32**	−0.37**	0.39**	0.23**	0.36**	−0.40**	0.30**	−0.29**	0.42**	0.36**	0.45**	**0.63**			
14. Vitality	3.80	0.95	0.29**	0.36**	−0.38**	0.41**	0.24**	0.27**	−0.28**	0.34**	−0.27**	0.38**	0.36**	0.35**	0.59**	**0.62**		
15. Dedication	3.86	0.94	0.34**	0.33**	−0.42**	0.29**	0.28**	0.41**	−0.36**	0.38**	−0.33**	0.40**	0.43**	0.41**	0.57**	0.53**	**0.59**	
16. Enthusiasm	3.84	0.90	0.38**	0.23**	−0.42**	0.36**	0.39**	0.37**	−0.41**	0.36**	−0.41**	0.40**	0.34**	0.34**	0.46**	0.48**	0.58**	**0.57**

The diagonal bold word is the square root of AVE; **p < 0.01, *p < 0.05.

## 4 Results

### 4.1 Common method bias test

As the data for all variables in the study were obtained from questionnaires, common method bias may arise (Tang and Wen, 2020). The Harman one-way test for common method bias was used, along with unrotated principal component factor analysis of the items of all variables, and it was found that there were 11 factors with eigenvalues greater than 1. Moreover, the first factor explained 28.315% of the total variance, which is much lower than the critical criterion of 50%, suggesting that the data of the present study did not suffer from a serious common method bias.

### 4.2 Multicollinearity test

The research takes the athlete engagement as the dependent variable, the coach’s leadership behavior, the athlete’s achievement goal orientation, and the athlete basic psychological needs as the independent variables to conduct the normality test and the standardization of each predictive variable. The results show that the adjusted *R*^2^ values are 0.309, 0.294, and 0.312, respectively, all exceeding 0.1. The corresponding VIF values are 1.129, 1.001, and 1.128, respectively, all of which are less than 5, indicating that there is no obvious multicollinearity between the independent variables of the model. The DW values of 1.786, 1.784, and 1.756 are close to 2, indicating that there is no significant serial correlation between the data, and the mediation effect test is conducted.

### 4.3 Descriptive statistics and correlation analysis

Table 1 presents the means, standard deviations, and Pearson’s correlation coefficients for the various dimensions of coach leadership behavior, achievement goal orientation, basic psychological needs, and athlete engagement. The results indicated significant positive correlations between athlete engagement and: (1) certain dimensions of coach leadership behavior (training guidance, democracy, social support, and positive feedback); (2) specific dimensions of achievement goal orientation (mastery-convergence and performance-convergence); and (3) dimensions of athletes’ basic psychological needs. Conversely, significant negative correlations were observed with authoritarian coaching behaviors and achievement goal orientations characterized by avoidance (mastery-avoidance and achievement-avoidance). Additionally, significant correlations were identified among the dimensions of coach leadership behavior, achievement goal orientation, and basic psychological needs. The significant correlations among the study variables established a solid foundation for subsequent mediation effect tests.

### 4.4 Analysis of demographic differences

According to the different personal background information of the research objects, the coaches were divided into different ages, genders, and coaching events, and the athletes were divided into different ages, genders, events, sports levels, and training years. The variance test was conducted on the leadership behaviors, total dimensions of achievement goal orientation, basic psychological needs, and the status quo and differences of athlete engagement of different groups of coaches. This paper explores the background factors that affect the coaches’ leadership behavior, the achievement goal orientation of high-level athletes, the basic psychological needs, and the athlete engagement. The results showed that there were significant differences in coach leadership behavior in gender (*p* < 0.05), but no significant differences in other aspects; there were significant differences in age, gender, and training years (*p* > 0.05), but no significant differences in other aspects (*p* < 0.05). The basic psychological needs of athletes had significant differences in training years and sports grades (*p* > 0.05), but no significant differences in other aspects (*p* < 0.05). There were significant differences in training years, sports grades, and sports events (*p* > 0.05), but no significant differences in other aspects (*p* < 0.05).

### 4.5 Construct and test the mediation model

#### 4.5.1 Construction of the mediation model

Significant correlations exist between athlete engagements, coaches’ leadership behaviors, achievement goal orientation, and basic psychological needs. These correlations meet the criteria for mediation effect, enabling the construction of three mediation models: M1, M2, and M3.The paths of model M1 have coach leadership behavior → basic psychological needs → athlete engagement, achievement goal orientation → basic psychological needs → athlete engagement; the paths of model M2 have coach leadership behavior → achievement goal orientation → athlete engagement, coach leadership behavior → achievement goal orientation → basic psychological needs → athlete engagement; the paths of model M3 have achievement goal orientation → coach leadership behavior → athlete engagement, achievement goal orientation → coaches’ leadership behavior → basic psychological needs → athlete engagement.

#### 4.5.2 Model fit test

According to the assumptions among variables, the structural equation model was constructed, and the maximum likelihood method was used to fit the path coefficient of the model. The model fitting index results were as follows: CMIN/DF = 1.456, GFI = 0.827, IFI = 0.947, NFI = 0.925, CFI = 0.947, RMSEA = 0.042, all the fitting indexes reached the acceptable level (Table 2).

**Table 2:** Model fitness fit indices.

Fits the index	CMIN/DF	GFI	AGFI	RMSEA	NFI	IFI	TLI	CFI
Standard figures	<3	>0.8	>0.8	>0.08	>0.8	>0.8	>0.8	>0.8
Measured value	1.456	0.827	0.812	0.036	0.823	0.937	0.933	0.936

#### 4.5.3 Tests and analyses of model M1

The direct effects of each variable were analyzed in regression analyses with athlete engagement as the dependent variable, basic psychological needs, coaching leadership behaviors, and achievement goal orientation as the independent variables, and with basic psychological needs as the dependent variable and coaching leadership behaviors and achievement goal orientation as the independent variables. The results of the study showed that there was a significant positive effect of basic psychological needs (*β* = 0.34, *p* < 0.01), coaches’ leadership behavior (*β* = 0.41, *p* < 0.01), and achievement goal orientation (*β* = 0.33, *p* < 0.01) on athlete engagement; and there was a significant positive effect of coaches’ leadership behavior (*β* = 0.36, *p* < 0.01), and achievement goal orientation (*β* = 0.33, *p* < 0.01) had a significant positive effect on athlete engagement.

**Figure 1:**
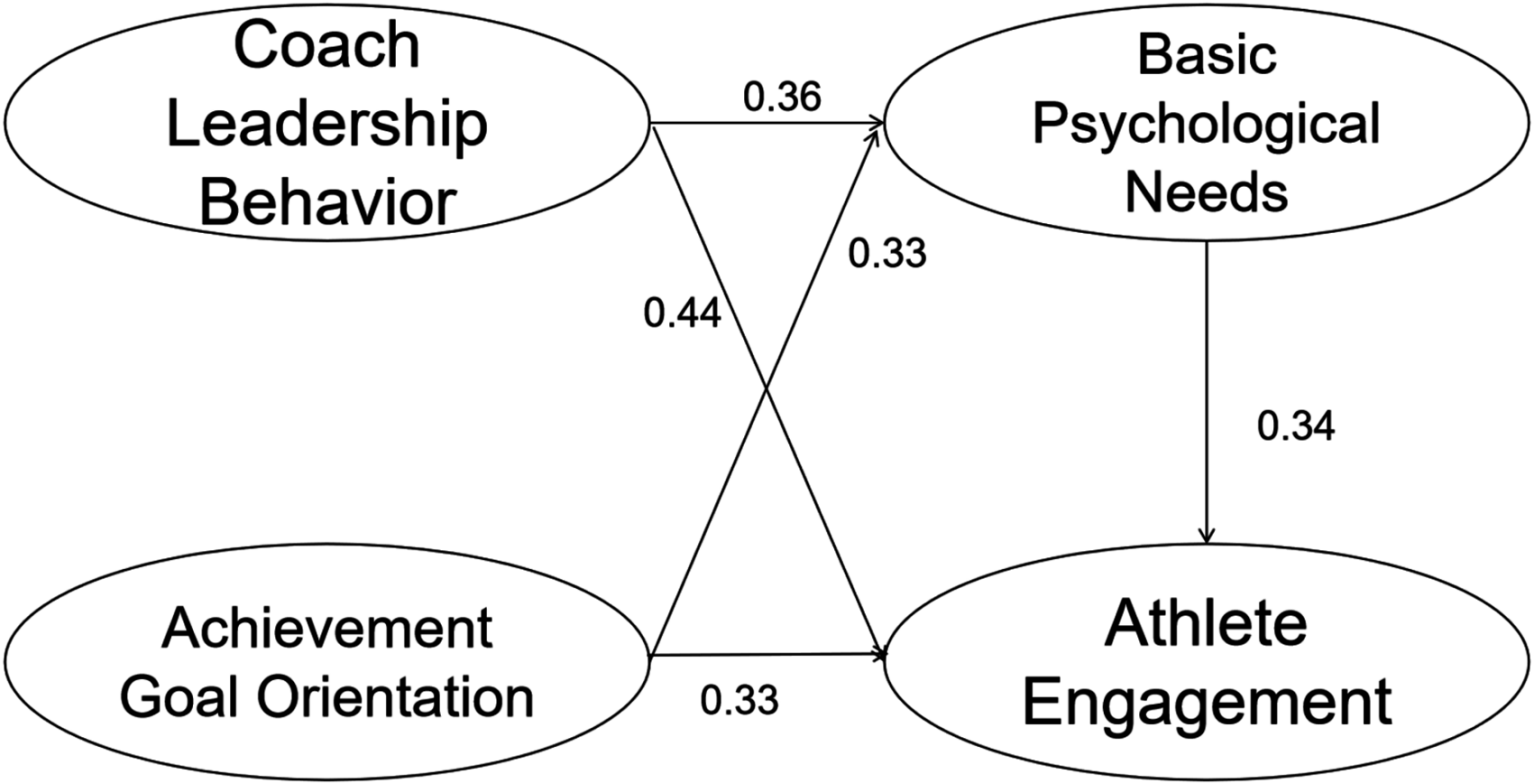
The double mediation model diagram of basic psychological needs in coach leadership behavior and achievement goal orientation.

For the test of mediating effect, the bias-corrected bootstrap 2,000 confidence interval estimation method was used to perform interval estimation, and the confidence level was set to 95% (Zhang and Kang, 2016). The double-intermediary model of basic psychological needs in coaches’ leadership behavior and achievement goal orientation was tested. The results (see Table 3) indicate that H1a-H1e, representing the five direct effect paths in the research hypotheses, exhibit significant *p*-values, with the endpoint values of the Bias-corrected and Percentile 95% confidence intervals being positive. Consequently, these five paths possess a significant direct effect. The hypotheses H1f-H1g represent two indirect paths with significant p-values, and their Bias-corrected and Percentile 95% confidence intervals exclude zero, suggesting that both indirect effects are significant. When comparing the indirect effects H1f and H1g, the confidence interval for their difference includes zero, indicating no significant difference between them. Based on the significance of both the direct effects (H1a-H1e) and indirect effects (H1f-H1g), it is evident that the mediating variables play a partial role in the relationship between the independent variables and the dependent variables. Specifically, basic psychological needs partially mediate between coaching leadership behaviors and athlete engagement, as well as between achievement goal orientation and athlete engagement. The path diagram derived from the above analysis, illustrating the impact of coaches’ leadership behaviors and achievement goal orientations on athlete engagement, is presented in Figure 1. In conclusion, Hypothesis 1 is supported.

**Table 3:** Analysis of the double mediating effect of basic psychological needs on coach leadership behavior and achievement goal orientation and athlete engagement.

Effect	Assumption route	Effect size	Magnitude of effect	SE	Bootstrapping			
					Bias-Corrected 95% CI		Percentile 95%CI	
Direct effect	H1a: BPN → AE	0.380**	19.07%	0.088	0.224	0.578	0.218	0.57
	H1b: CLB → AE	0.358**	17.97%	0.086	0.195	0.536	0.2	0.54
	H1c: AGO→AE	0.302**	15.15%	0.078	0.15	0.453	0.146	0.449
	H1d: CLB → BPN	0.351**	17.61%	0.073	0.211	0.501	0.207	0.498
	H1e: AGO→BPN	0.332**	16.66%	0.07	0.21	0.481	0.207	0.476
Intermediary effect	H1f: CLB → BPN → AE	0.134**	6.72%	0.041	0.073	0.24	0.067	0.227
	H1g:AGO→BPN → AE	0.136**	6.82%	0.04	0.075	0.241	0.07	0.228
Poor indirect effect	H1f-H1g	−0.002		0.049	−0.096	0.108	−0.099	0.104

AGO, achievement goal orientation; AE, athlete engagement; CLB, coach leadership behavior; BPN, basic psychological needs; **p < 0.01, *p < 0.05.

#### 4.5.4 Tests and analyses of model M2

The dependent variable relationships are the same, so the regression analyses of the M2 variable relationships are the same as for M1.

**Figure 2:**
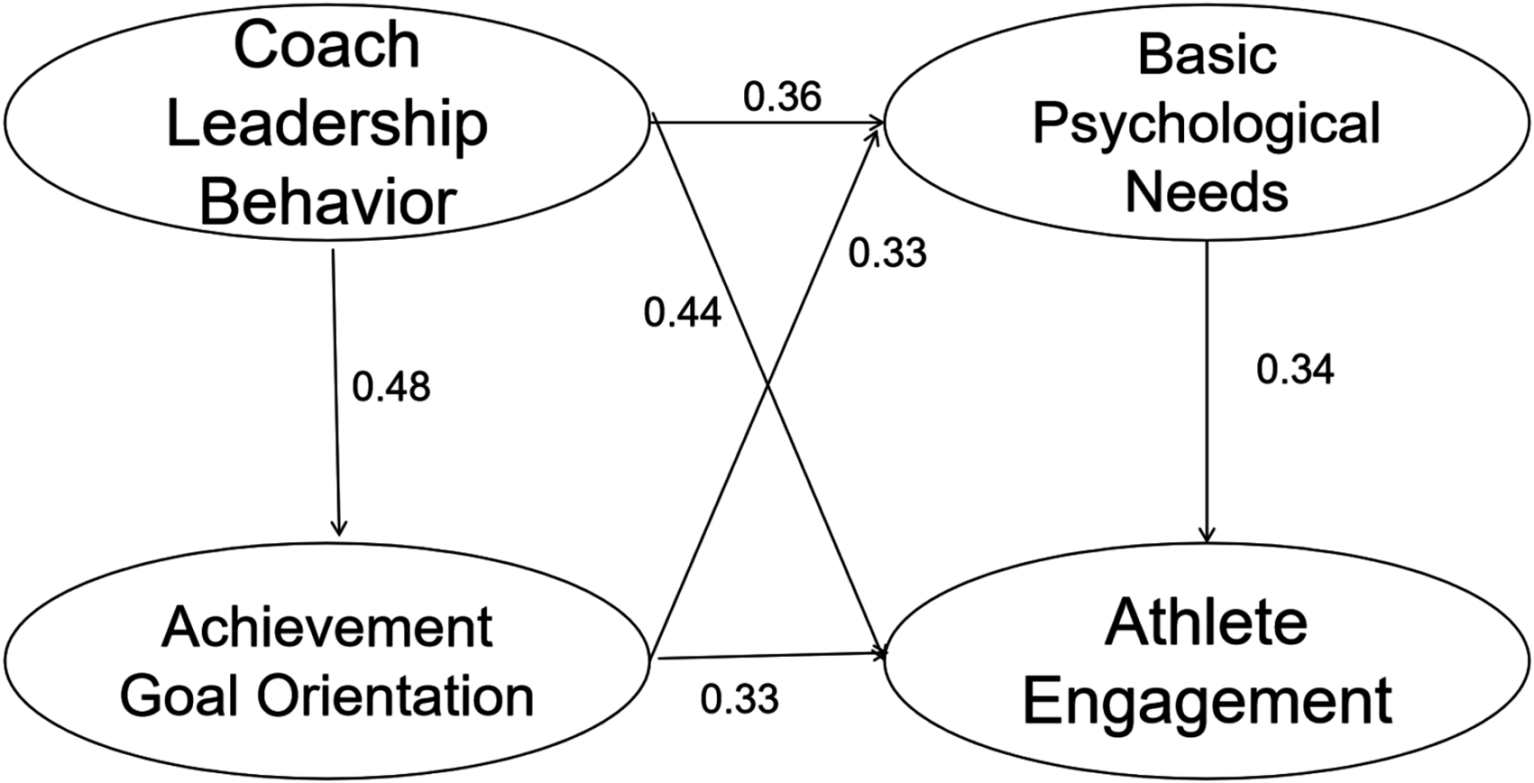
The chain mediation model of achievement goal orientation and basic psychological needs between coach leadership behavior and athlete engagement.

The effect values and significance test results pertaining to the chain mediation involving achievement goal orientation and basic psychological needs are presented in Table 4. The research hypotheses H2a-H2e correspond to five direct effect paths with statistically significant *p*-values. The endpoint values of the bias-corrected and percentile 95% confidence intervals for these paths are positive, confirming a significant direct effect. This supports the hypotheses H2a-H2e proposed in this study and aligns with the findings of the M1 model study. The research hypotheses H2f-H2i represent four indirect paths, all with statistically significant *p*-values. The bias-corrected and percentile 95% confidence intervals for these paths do not include zero, indicating the establishment of an indirect effect. A comparison was conducted between the indirect effects of H2f and H2h, and H2h and H2i, specifically examining the differences in the effects mediated by coaches’ leadership behavior and achievement goal orientation, respectively. Additionally, a comparison was made between the chain mediator effect and the simple mediator effect on athlete engagement. Using the Bootstrap method, the effect size difference between H2f and H2h was found to be −0.038, while the effect size difference between H2h and H2i was 0.100. However, the bias-corrected and percentile 95% confidence intervals for these differences included zero, indicating that neither difference was statistically significant. Given the significant direct effects of H2a-H2e and indirect effects of H2f-H2i, it is evident that the mediating variable plays a partial role in the relationship between the independent and dependent variables. Specifically, basic psychological needs mediate partially between coaches’ leadership behaviors and athlete engagement, as well as between achievement goal orientations and athlete engagement. Furthermore, achievement goal orientations play a partial role in the mediation chain linking coaches’ leadership behaviors to athlete engagement. Therefore, the chain-mediating role of achievement goal orientation and basic psychological needs in the relationship between coach leadership behaviors and athlete engagement is established. The path diagram resulting from the aforementioned analyses is depicted in Figure 2. In conclusion, Hypothesis 2 is confirmed as valid.

**Table 4:** Chain mediation effect analysis of achievement goal orientation and basic psychological needs on coach leadership behavior and athlete engagement.

Effect	Assumption route	Effect size	Magnitude of effect	SE	Bootstrapping			
					Bias-Corrected 95% CI		Percentile 95%CI	
Direct effect	H2a: BPN → AE	0.380**	16.99%	0.088	0.224	0.578	0.218	0.570
	H2b: CLB → AE	0.358**	16.00%	0.086	0.195	0.536	0.200	0.540
	H2c: AGO→AE	0.302**	13.50%	0.078	0.150	0.453	0.146	0.449
	H2d: CLB → BPN	0.351**	15.69%	0.073	0.211	0.501	0.207	0.498
	H2e: AGO→BPN	0.332**	14.84%	0.070	0.210	0.481	0.207	0.476
Intermediary effect	H2f: CLB → BPN → AE	0.134**	5.99%	0.041	0.073	0.240	0.067	0.227
	H2g: AGO→BPN → AE	0.136**	6.07%	0.040	0.075	0.241	0.070	0.228
	H2h: CLB → AGO→AE	0.172**	7.69%	0.050	0.086	0.285	0.081	0.275
	H2i: CLB → AGO→BPN → AE	0.072**	3.22%	0.025	0.035	0.136	0.032	0.129
Aggregate effect		2.237	100%					
Poor indirect effect	H2f-H2h	−0.038		0.065	−0.165	0.098	−0.167	0.097
	H2h-H2i	0.100		0.057	−0.017	0.213	−0.019	0.212

AGO, achievement goal orientation; AE, athlete engagement; CLB, coach leadership behavior; BPN, basic psychological needs; **p < 0.01, *p < 0.05.

#### 4.5.5 Tests and analyses of model M3

The dependent variable relationships are the same, so the regression analyses of the M3 variable relationships are the same as M1.

**Figure 3:**
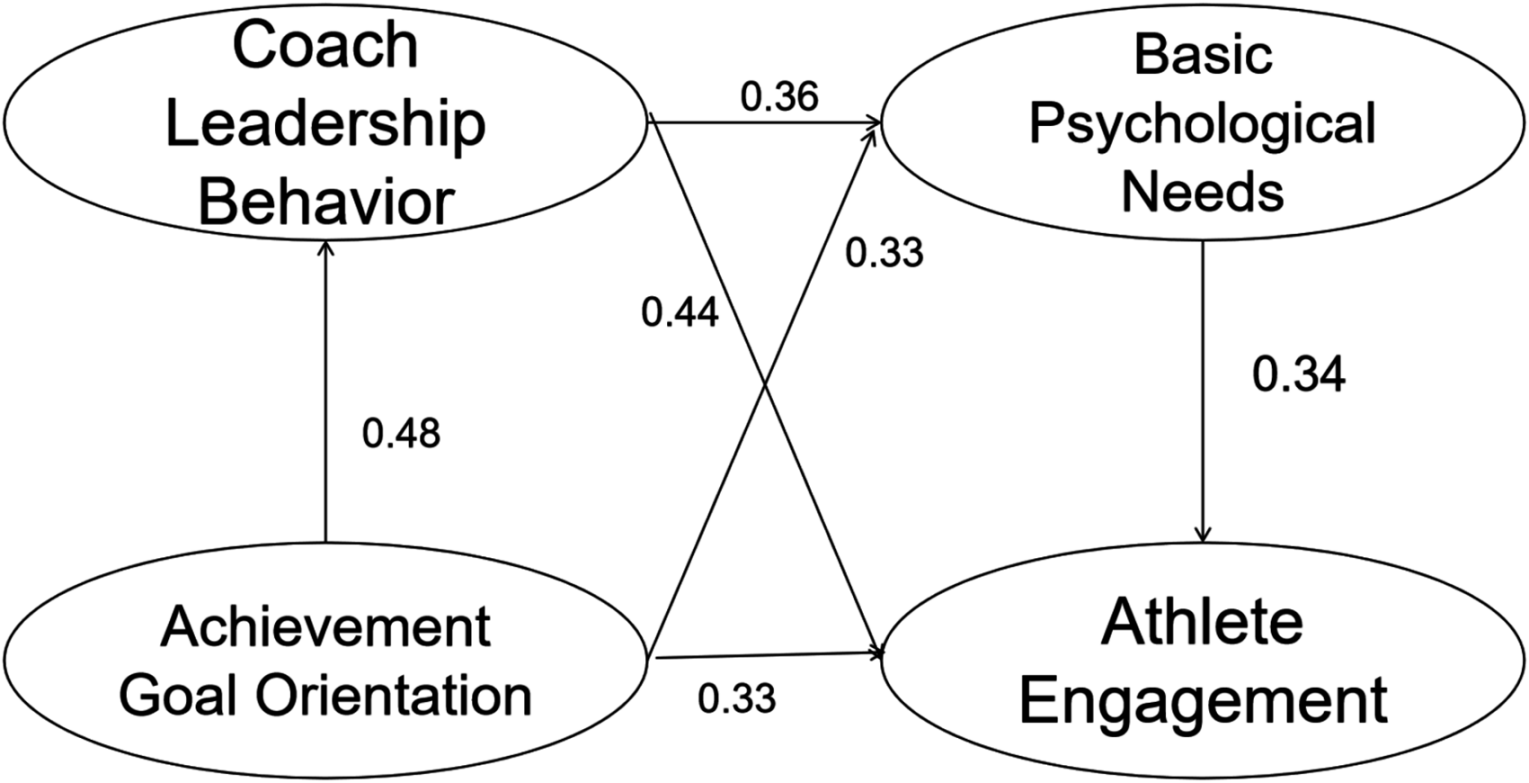
The chain mediation model of coach leadership behavior and basic psychological needs in achievement goal orientation and athlete engagement.

The effect values and significance test results pertaining to the chain mediation between coach leadership behaviors and basic psychological needs are presented in Table 5. The research hypotheses H3a-H3e represent five direct effect paths with significant *p*-values and positive endpoint values within the Bias-corrected and Percentile 95% confidence intervals, supporting the hypotheses proposed in this study and aligning with the findings of the M1 and M2 model studies. The research hypotheses H3f-H3i represent four indirect paths with significant *p*-values and Bias-corrected and Percentile 95% confidence intervals excluding zero, indicating that the indirect effects are present. The effects of H3g and H3h were compared, with basic psychological needs and coaches’ leadership behavior serving as mediating variables, respectively. The comparison between the chained and simple mediation effects on athlete engagement (H3g vs. H3h) was conducted using the Bootstrap method. The difference effect size was −0.006, with both Bias-corrected and Percentile 95% confidence intervals including zero, indicating no significant difference between the two specific mediating effects. However, for another comparison, the effect size of the difference between H3g and H3h was 0.089, with both confidence intervals excluding zero, suggesting a significant difference, where the indirect effect of coaching leadership behavior as the mediator was slightly greater than that of the chained mediator effect involving both coach leadership behavior and basic psychological needs. Based on the significant results of both H3a-H3e direct effects and H3f-H3i indirect effects, it is evident that the mediating variable plays a partial role in the relationship between the independent and dependent variables. Specifically, basic psychological needs mediate partially between coach leadership behaviors and athlete engagement, as well as between achievement goal orientations and athlete engagement. Furthermore, the chained mediating role of achievement goal orientations in the relationship between coach leadership behavior and athlete engagement is established, as well as the chained mediating role of coach leadership behavior and basic psychological needs in the context of achievement goal orientation and athlete engagement. The path diagram derived from the above analyses is presented in Figure 3. In conclusion, Hypothesis 3 is supported.

**Table 5:** Chain mediation effect analysis of coach leadership behavior and basic psychological needs on achievement goal orientation and athlete engagement.

Effect	Assumption route	Effect size	Magnitude of effect	SE	Bootstrapping			
					Bias-Corrected 95% CI		Percentile 95%CI	
Direct effect	H2a: BPN → AE	0.380**	17.36%	0.088	0.224	0.578	0.218	0.570
	H2b: CLB → AE	0.358**	16.35%	0.086	0.195	0.536	0.200	0.540
	H2c: AGO→AE	0.302**	13.80%	0.078	0.150	0.453	0.146	0.449
	H2d: CLB → BPN	0.351**	16.03%	0.073	0.211	0.501	0.207	0.498
	H2e: AGO→BPN	0.332**	15.17%	0.070	0.210	0.481	0.207	0.476
Intermediary effect	H2f: CLB → BPN → AE	0.134**	6.12%	0.041	0.073	0.240	0.067	0.227
	H2g: AGO→BPN → AE	0.136**	6.21%	0.040	0.075	0.241	0.070	0.228
	H3h: AGO→CLB → AE	0.143**	6.53%	0.039	0.077	0.232	0.075	0.228
	H3i: AGO→CLB → BPN → AE	0.053**	2.42%	0.019	0.028	0.106	0.024	0.097
Aggregate effect		2.189	100%					
Poor indirect effect	H2g-H2h	−0.006		0.039	−0.089	0.067	−0.089	0.067
	H2h-H2i	0.089**		0.044	0.003	0.182	0.004	0.182

AGO, achievement goal orientation; AE, athlete engagement; CLB, coach leadership behavior; BPN, basic psychological needs; **p < 0.01, *p < 0.05.

#### 4.5.6 A comparative study of the magnitude of the mediating role of coach leadership behavior and achievement goal orientation between athlete engagement

In order to explore the relationship between basic psychological needs, coach leadership behavior, and achievement goal orientation, and athlete engagement, based on the results of related research, model testing was carried out in this study by replacing the dependent and mediating variables respectively, and three models were established as follows, which are detailed in Table 6.

**Table 6:** Summary of mediation models between basic psychological needs and coach leadership behavior and achievement goal orientation across athlete engagement.

	Model effect value	Independent variable	Intermediary variable	Dependent variable
Model 1	1.993	Coach leadership behavior Achievement goal orientation	Basic psychological needs	Athlete engagement
Model 2	2.237	Coach leadership behavior	Achievement goal orientation Basic psychological needs	
Model 3	2.189	Achievement goal orientation	Coach leadership behavior Basic psychological needs	

**Figure 4:**
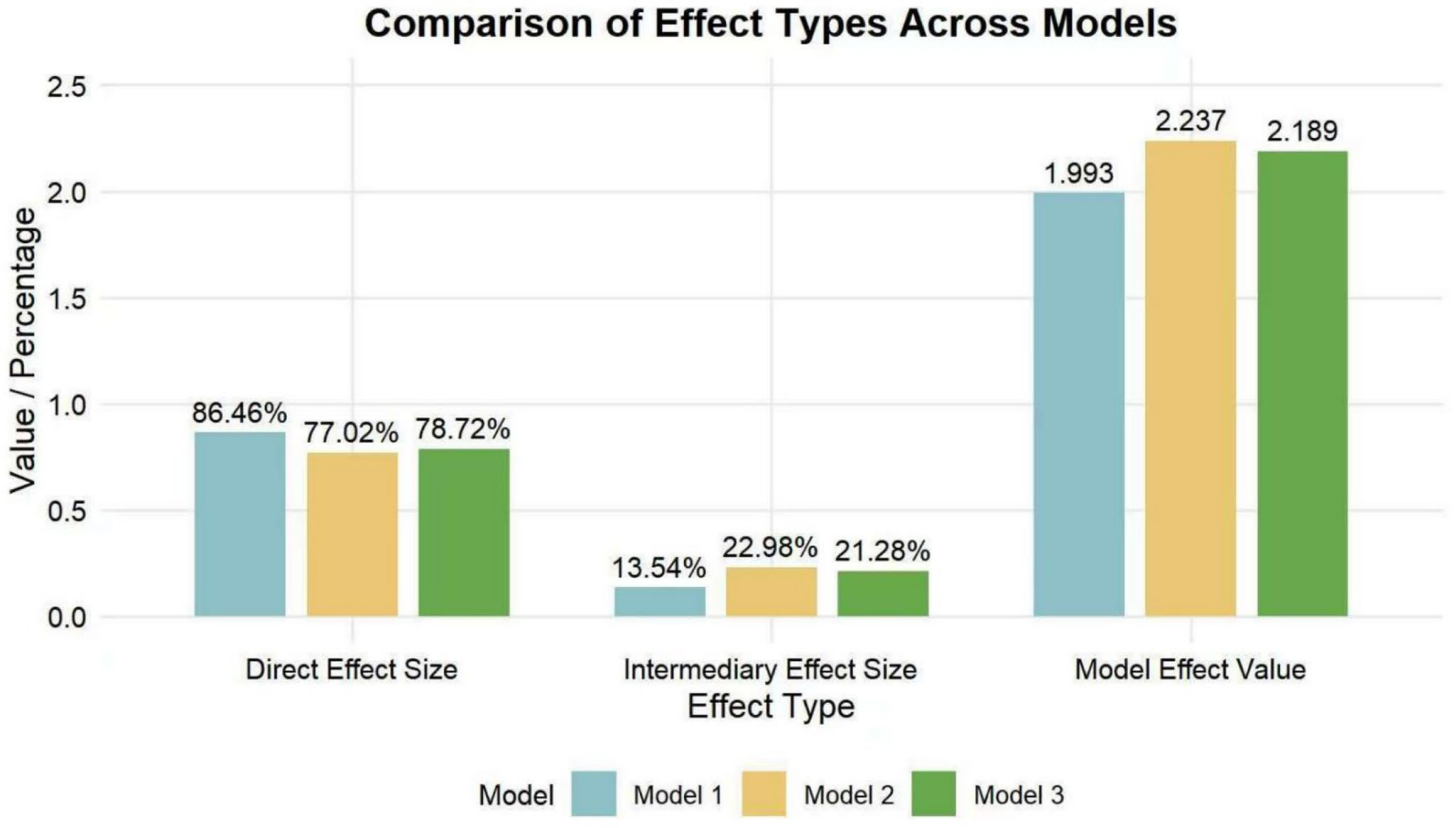
Comparison of the mediating effects of basic psychological needs, coach leadership behavior, and achievement goal orientation on athlete engagement.

After the model test, the mediating effect of all three models was established, and the direct effect size, mediating effect size, and total effect value of each model were compared, and the results are shown in Table 7 and Figure 4.

**Table 7:** Summary of mediating effects between basic psychological needs and coach leadership behavior and achievement goal orientation on athlete engagement.

	Model effect value	Direct effect size	Intermediate effect size	Independent variable	Intermediary variable	Dependent variable
Model 1	1.993	86.46%	13.54%	Coach leadership behavior Achievement goal orientation	Basic psychological needs	Athlete engagement
Model 2	2.237	77.02%	22.98%	Coach leadership behavior	Achievement goal orientation Basic psychological needs	
Model 3	2.189	78.72%	21.28%	Achievement goal orientation	Coach leadership behavior Basic psychological needs	

Figure 4 shows that the effect value of the influence of coaching leadership behavior as the independent variable on athlete engagement is the largest, which shows the important influence of different leadership behaviors of coaches on athlete engagement. Further comparison of the mediating effects in the three models shows that the mediating effect in model 2 accounts for the highest proportion of the total effect value, which indicates that coaches’ leadership behavior is most affected by achievement goal orientation and basic psychological needs, i.e., coach leadership behavior has the strongest plasticity, and different coach leadership behavior should be paid attention to in order to enhance athlete engagement.

## 5 Discussion

### 5.1 The direct role of coach leadership behavior and achievement goal orientation on athlete engagement

A direct effect test of the coach leadership behavior and achievement goal orientation-athlete engagement model through constructive structural equations found that both coach leadership behavior and achievement goal orientation had a direct effect on athlete engagement, which is consistent with many studies.

Coaches are the direct leaders of athletes and are the most closely related working partners. Through different behavioral approaches of coaches, as external motivation, athletes can be motivated to actively engage in training and competitions and enhance their sport performance. Reynders et al. (2019) suggest that coaches can be more skilled in adopting demand-supportive coaching behaviors, which are conducive to the autonomous motivation and engagement of athletes; Ye Lü et al. and Nian Shi et al. suggest that social supportive behaviors of coaches can promote the athlete’s sense of participation in the sport training tasks and actively engage in training and competition (Ye et al., 2016c; Nian, 2014). The findings of Cai and Wu (2014) also showed that coaches’ training instruction behaviors can have a motivating, internalizing effect on athletes. In a study by Gao et al. (2021), it was shown that democratic, authoritarian, and rewarding behaviors in coach leadership behaviors do not have a direct effect on athlete engagement but can indirectly have an effect on athlete engagement by influencing the coach-athlete relationship. In addition, coach leadership behavior plays a crucial role in the development of the coach-athlete relationship and significantly improves athlete involvement. Therefore, coaches can consider building good relationships rather than just applying leadership skills, as this can significantly improve athletes’ emotional resilience and mental health and reduce athlete burnout (Duhaylungsod et al., 2024).

Achievement goal orientation is the individual’s perception of the purpose or reason for engaging in an achievement task and the individual’s ability to complete this task or reach a standard of success. Achievement goals have a direct effect on high-level athlete engagement, with convergent goal orientations positively predicting athlete engagement and avoidant goal orientations negatively predicting athlete engagement, which is consistent with existing research (Pintrich, 2000). Psychology emphasizes (Conroy et al., 2010) that motivation is an internal force that triggers an individual’ s activity cabinet, sustains and maintains the activity towards a certain goal, and is the basic source of stimulating individual behavior. As a concept in the theory of individual motivation, achievement goals influence the level of athlete engagement to some extent.

### 5.2 The mediating role of basic psychological needs between coach leadership behavior and achievement goal orientation and athlete engagement

#### 5.2.1 The mediating role of basic psychological needs

Basic psychological needs were found to play a dual mediating role between coach leadership behavior and athlete engagement and achievement goal orientation and athlete engagement. In the M1 model, the hypotheses of H1a-H1e were established and the direct effects were significant. In addition, in the prediction study, it was also proved that coach leadership behavior and achievement goal orientation had significant predictions on basic psychological needs, which indicated that coach leadership behavior and achievement goal orientation were important facilitators of basic psychological needs. H1f mediating effect path “coach leadership behavior → basic psychological needs → athlete engagement” and H1g mediating effect path “achievement goal orientation → basic psychological needs → athlete engagement” were significant. Therefore, coach leadership behavior can influence basic psychological needs and promote athlete engagement because individuals’ basic psychological needs are affected by the external environment (Li et al., 2019). The coach guides, trains, and prepares for the competition according to the technical requirements of the athlete, combines theory and practice, and its training and guidance behavior can help meet the ability needs of the athlete. Autocratic behavior is detrimental to athletes’ sense of self-efficacy (Sari and Bayazit, 2017). Therefore, it is suggested that coaches avoid autocratic behavior and let athletes participate in the decision-making process through democratic behavior so that athletes can have more self-concern, self-regulation, and a sense of connection with others, and promote athletes’ need for autonomy, which then further promotes athlete engagement in sport. The human organism is constantly striving for a sense of competence, self-determination, and belonging with others, and to satisfy the 3 basic psychological needs of autonomy, competence, and relatedness, and athletes are no exception. This process of motivation is seen as intrinsic, but it can be fostered in the environment through autonomy support, competence support, and interpersonal support functions. By providing support in training and creating an environment for athletes to train autonomously with features such as autonomous choice and emotional understanding, coaches can not only increase athletes’ sense of belonging to the sports team but also enhance the level of athletes’ engagement (Zhu et al., 2011). Basic psychological needs play a mediating role between achievement goal orientation and athlete engagement, i.e., achievement goal orientation can directly predict athlete engagement and can also influence athlete engagement through basic psychological needs. This is consistent with the findings of a previous study (Vlachopoulos and Michailidou, 2006) that when athletes are in a better state of mastering goals and performance goals, the basic psychological satisfaction of athletes will be improved and promoted. Conversely, the satisfaction of athletes’ ability needs and relationship needs can enhance athletes’ task orientation (Sari, 2015), and the corresponding enthusiasm of athletes will be higher.

#### 5.2.2 Sequential mediation of basic psychological needs and achievement goal orientation

Basic psychological needs and achievement goal orientation have a chain-mediated role in the influence of coach leadership behavior on athlete engagement. Therefore, the significance of achievement goal orientation in the chain mediation model is not only reflected in its positive influence on athlete engagement but also affects the role of individual basic psychological needs satisfaction in influencing athlete engagement. However, the mediation effect of the chain mediation path “coach leadership behavior → achievement goal orientation → basic psychological needs → athlete engagement” is smaller than that of the mediation path “coach leadership behavior → achievement goal orientation → athlete engagement.” This may be due to the fact that the mastery-avoidance and performance-avoidance goal orientations of the achievement goal orientations in this study weakened the satisfaction of individuals’ basic psychological needs, which in turn reduced the positive effect on athlete engagement. It has been shown that as the perception of autonomous support increases, so does the positive motivational response, especially when the athlete’s perceived level of controlling behavior is relatively low, and the most positive motivational outcome is associated with higher autonomous support and lower perception of controlling behavior (Amorose and Anderson-Butcher, 2015). In this regard, coaches can relieve athletes’ negative emotions such as pressure and depression through mindfulness support therapy and democratic support behavior, enhance athletes’ positive motivation level, avoid weakening the satisfaction of basic psychological needs, and improve athletes’ performance (Solmaz and Yarayan, 2025).

#### 5.2.3 Sequential mediation of basic psychological needs and coach leadership behavior

The chain mediation effect of “coach leadership behavior → basic psychological needs” between achievement goal orientation and athlete engagement was significant, which verified the hypothesis H3. The chain mediating effect accounted for 2.42% of the total effect, reaching the significant level, indicating that among the mediating effects of basic psychological needs affecting athlete engagement, there is also a causal chain transformation path, that is, a part of achievement goal orientation first goes through the internalization of leadership behavior and then influences athlete engagement through the transformation of basic psychological needs. The results of this study indicate that athlete engagement is influenced by external and individual factors, and the behavior displayed by coaches during athletes’ training and competition will have an impact on athletes’ motivation, emotions, psychology, and behavior. In the process of the formation of athletes’ achievement motivation orientation, athletes’ goal orientation interacts closely with coaches’ leadership styles, and coaches’ feedback styles have an impact on athletes’ achievement motivation orientation (Wang and Zhang, 2017). In addition, the satisfaction of athletes’ basic psychological needs is associated with higher levels of intrinsic training motivation and with coaching behaviors that support athletes’ autonomy (Inguglia et al., 2023). The coach’s autonomy support can significantly affect the athletes’ autonomy needs, ability needs, and relationship needs, and the satisfaction of these three psychological needs will affect the athletes’ autonomous motivation, and then affect athlete engagement (Lourenço et al., 2022). Thus, rational and effective training instruction as well as the satisfaction of basic psychological needs is good medicine to enhance athlete engagement. Coaches are advised to combine autonomy-supportive behaviors (e.g., involving athletes in goal setting) with cognitive-behavioral techniques (e.g., motivational interviewing); combining democratic decision-making with reflective issues can simultaneously satisfy the need for autonomy and align training with the athlete’s intrinsic goals, thus amplifying the mediating role of basic psychological needs.

#### 5.2.4 Differences in the role of intermediaries

The mediation effect holds in all three models, and comparing the effect values, it is known that the chained mediation with coach leadership behavior as the independent variable is stronger than the chained effect with achievement goal orientation as the starting point, and the mediation influence is higher. It can be seen that coaches, as the direct leaders of athletes and achievement goal orientation, as individual factors, have a very important influence on athlete engagement in sports, in which coach leadership behavior is the most malleable. Coach leadership behaviors are social supportive behaviors, and with these supportive behaviors, athletes are more willing to cooperate with coaches and change from passive performers to active participants and decision makers. This satisfaction of autonomy further enhances athletes’ motivation to participate and inspires athletes to improve their level of athlete engagement. The coach’s positive behavior, failure tolerance, and encouragement to try help build a task-oriented incentive atmosphere, and realize the internalization of external motivation through the transfer of athletes, and athletes show positive behavior orientation (Huang et al., 2021). In the chain-mediated analysis, the influence mechanism of coach leadership behavior as an external factor and achievement goal orientation as an internal factor on athlete engagement was also further refined. Although the influence effect of chain mediation accounts for a relatively low percentage, it still explains to a certain extent the transmission psychological mechanism of the influence of coach leadership behavior and achievement goal orientation on athlete engagement and provides empirical evidence for a clear understanding of the psychological influences on athlete engagement. Although this study verifies the influence mechanism of Chinese athletes’ participation, its cultural particularity requires careful interpretation. The background of competitive sports with Chinese characteristics and hierarchical social norms may have an impact on the leadership behavior of coaches and the psychological mechanism of athletes. For example, Chinese athletes have become accustomed to a highly disciplined sports environment and formal relationships with coaches (Lenartowicz, 2023), which may amplify the role of transformational or paternalistic leadership styles in meeting athletes’ psychological needs. In the individualistic Western context, athletes’ evaluations of coaches are pro-autonomy, and their motivations are self-determined (Johannes et al., 2019). In addition, the goal orientation of Chinese athletes is often influenced by family and social expectations, while the motivation of Western athletes is more personal. This comparison suggests that the intensity and variability of the mediating pathways identified in the model may vary from culture to culture, and future investigations should be expanded to discuss whether these results apply to athletes from other cultural backgrounds.

#### 5.2.5 Limitations and research perspectives

This study used cross-sectional data to explore the relationships between coaches’ leadership behavior, basic psychological needs, achievement goal orientation, and athlete engagement, but it was difficult to determine the causal relationship. Athlete engagement is dynamic in nature and needs longitudinal studies to explore its formation mechanism. Secondly, in the investigation process of this study, the sample selection scope is relatively limited, which makes it difficult to represent all high-level athletes. There are great differences in sports culture, training mode, and development level in different regions, which will lead to certain limitations in the research results. Follow-up studies should further expand the sample coverage to cover high-level athletes from different regions and different sports characteristics across the country, as well as the country of different cultural values, so as to obtain more general research conclusions and comprehensively and deeply understand the influence of various factors on athlete engagement. This study did not discuss the dimensions of each variable separately, and the model limitations have led to the exclusion of other influencing factors of athlete engagement, such as personality traits, psychological fatigue, parental support, and environmental factors such as team cohesion. Therefore, in future research, it should be further refined by integrating and including new variables from multiple theoretical perspectives to explore the formation mechanism model of athlete engagement. Athlete engagement, as a positive cognitive and emotional experience, is often treated as a dependent variable and predicted by other variables. However, some studies have shown that athlete engagement can not only be influenced by other predictor variables but also serve as a predictor variable to affect other dependent variables. Early studies found that athlete engagement could predict an athlete’s state of fluid experience (Hodge et al., 2009) and satisfaction with athletic performance (Ye et al., 2016c). As a positive predictor variable, the predictive ability of athlete engagement needs to be further confirmed by research. Meanwhile, experimental intervention studies should be conducted to bridge the gap between theory and practice.

## 6 Conclusion

Basic psychological needs, coach leadership behaviors, and achievement goal orientations all predicted athlete engagement, and all four were correlated with each other. The total dimension of basic psychological needs and coach leadership behaviors of training and instruction, democratic behaviors, social supportive behaviors, and positive feedback behaviors, as well as mastery-convergent and achievement-convergent goal orientations, significantly and positively predicted athlete engagement, whereas authoritarian behaviors and mastery-avoidant and achievement-avoidant goal orientations significantly and negatively predicted athlete engagement. Basic psychological needs mediated both between coach leadership behaviors and athlete engagement and between achievement-goal orientations and athlete engagement; there were dual chained mediation mechanisms between coach leadership behaviors and achievement-goal orientations and between basic psychological needs and athlete engagement, which were compared by model tests and model differences. The chain mediation model based on coaches’ leadership behavior has the largest effect, which indicates that optimizing coaches’ leadership behavior in actual training is of great significance to improving athletes’ participation level. The mediation model effect established in this study reveals the mechanism of athletes’ basic psychological needs, coaches’ leadership behavior, and athletes’ achievement goal orientation on athletes’ engagement to a certain extent, which has certain reference value for the promotion of athletes’ engagement.

## Data Availability

The original contributions presented in the study are included in the article/supplementary material, further inquiries can be directed to the corresponding author.
